# Effectiveness of jejunostomy for enteral nutrition during complete thoracoscopic and laparoscopic Ivor-Lewis esophagectomy in thoracic segment esophageal carcinoma

**DOI:** 10.1186/s13019-020-01162-7

**Published:** 2020-06-17

**Authors:** Jieyong Tian, Xinyu Mei, Mingfa Guo, Ran Xiong, Xiangxiang Sun

**Affiliations:** Department of Thoracic Surgery, The First Affiliated Hospital of USTC, Division of Life Sciences and Medicine, University of Science and Technology of China, Hefei, 230036 Anhui China

**Keywords:** Esophageal neoplasm, Esophagectomy, Enteral nutrition, Jejunostomy, Nasointestinal tube, Ivor-Lewis, McKowen

## Abstract

**Background:**

Although jejunostomy is widely used in complete thoracoscopic and laparoscopic minimally invasive Ivor-Lewis esophagectomy, its clinical effectiveness remains undefined. This study aimed to assess the therapeutic and side effects of jejunostomy in patients undergoing Ivor-Lewis esophagectomy for thoracic segment esophageal carcinoma.

**Methods:**

A total of 1400 patients with esophageal carcinoma who underwent minimally invasive esophagectomy in the Thoracic Surgery of our hospital from 2015 to 2018 were retrospectively evaluated. Of these, 356 and 1044 were treated with nasojejunal feeding tubes (Nasojejunal group) and by jejunostomy (Jejunostomy group), respectively. Clinicopathologic factors, postoperative complications and tubule-related complications between the two groups were compared.

**Results:**

Both groups were well-balanced for clinicopathological data, except tumor location, which was significantly different (*P* < 0.001). Operation time (208.8 ± 53.5 min vs. 218.1 ± 43.2 min) was shorter in the Jejunostomy group compared with the Nasojejunal group, while intraoperative (26.6 ± 10.4 min vs 18.4 ± 9.1 min) and postoperative (38.6 ± 6.9 min vs 18.5 ± 7.6 min) indwelling times of nutrition tubes were prolonged (all *P* < 0.05). Postoperative pulmonary infection (17.0% vs 22.2%), incision infection (0.2% vs 1.1%), nutrient tube slippage (0.2% vs 5.1%) and nutrient reflux 1 (0.1% vs 5.6%) rates were reduced in the Jejunostomy group compared with the Nasojejunal group (P < 0.05). Meanwhile, ileus rates perioperatively (1.7% vs 0.3%) and at 3 postoperative months (1.7% vs 0.3%) were both higher in the Jejunostomy group compared with the Nasojejunal group.

**Conclusions:**

Jejunostomy is a reliable enteral nutrition method in Ivor-Lewis esophagectomy for thoracic segment esophageal carcinoma.

## Background

Esophageal cancer, an extremely aggressive malignancy, ranks numbers 6 and 8 among the deadliest and most common cancers worldwide, respectively; its incidence exceeds 100 cases/100000 person-years in some regions, with a 5-year survival rate approximating 15–25% [1]. Esophageal cancer can be divided into squamous cell carcinoma and adenocarcinoma subtypes [2]. Risk factors include gender, race, smoking, drinking, diet, genetics, obesity, drug use, and a history of mediastinal radiation, gastroesophageal reflux disease or Barrett’s esophagus [1, 3].

At present, surgery is the main treatment option for resectable esophageal carcinoma [4, 5]. The National Comprehensive Cancer Network (NCCN) provides guidelines for treating esophageal cancer, and surgical options encompass local mucosal resection and ablation therapies and esophagectomy [6]. Postoperatively, the incidence of anemia and hypoproteinemia are high, because patients usually have long-term eating difficulties, especially the elderly [7]. Therefore, the postoperative nutritional status of patients with esophageal carcinoma is considered an important factor in preventing postoperative complications and ensuring the success of surgical treatment [8, 9].

Given its advantages of comprehensive nutrition, easy initiation, and satisfying physiological requirements for nutrient absorption in the human body, enteral nutrition (EN) has been applied by most clinicians [10–12]. Currently, the most common EN methods employ nasointestinal and jejunostomy tubes. Clinical application of the nasointestinal tube is common, and its efficacy is widely recognized, but there are shortcomings, including poor comfort and easy slippage during tube indwelling, as well as poor tolerance [13]. Jejunostomy is mainly used in complete thoracoscopic and laparoscopic minimally invasive Ivor-Lewis esophagectomy, which can significantly improve the patients’ degree of comfort and portability due to the nutrition tube being located in the abdomen, thus improving the quality of life during indwelling and in postoperative EN support [14]. However, some scholars believe that jejunostomy is an invasive operation, which increases surgical difficulty and trauma, with more tubule-related complications [15].

Since January 2015, our center has routinely used laparoscopic jejunostomy for minimally invasive Ivor-Lewis esophagectomy, and routine intraoperative indwelling of nasointestinal tubes for minimally invasive McKowen esophagectomy. Despite the wide application of jejunostomy, its clinical effectiveness remains undefined. Therefore, the present retrospective study aimed to assess the therapeutic and undesired effects of jejunostomy in individuals administered Ivor-Lewis esophagectomy for thoracic segment esophageal carcinoma. The clinical data of 1400 patients with esophageal carcinoma were assessed, and various parameters in both EN methods were compared to investigate the effectiveness and reliability of jejunostomy in complete thoracoscopic and laparoscopic minimally invasive Ivor-Lewis esophagectomy.

## Methods

### Study design and patients

**Fig. 1:**
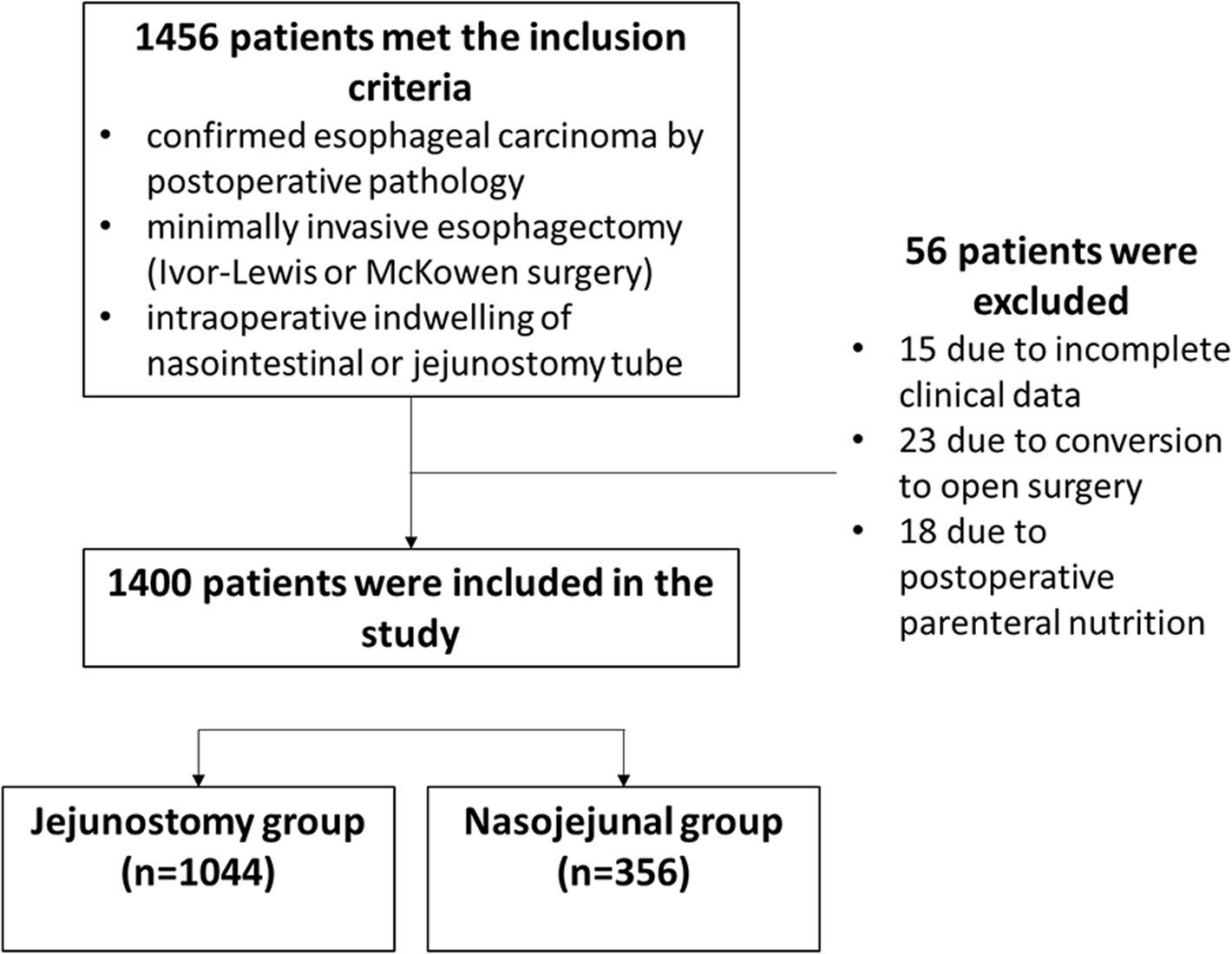
Flowchart showing inclusion of patients in the study

This was a retrospective study conducted at the department of Thoracic Surgery, the First Affiliated Hospital of University of Science and Technology of China, from January 2015 to June 2018. The surgical approach was usually chosen according to tumor location. Tumor staging in esophageal cancer patients was performed using the AJCC 8th edition of TNM staging system [16]. Cases were consecutively enrolled in the study and those with upper esophageal cancer usually underwent McKeown surgery, while those with middle or lower esophageal cancer could undergo either Ivor-Lewis or McKeown surgery. The patients undergoing minimally invasive Ivor-Lewis esophagectomy and McKeown surgery received routine tube feeding via jejunostomy and indwelling nasointestinal tubes, respectively. Accordingly, the patients were assigned to the jejunostomy and nasojejunal groups (Fig. 1).

Inclusion criteria were: 1) confirmed esophageal carcinoma by postoperative pathology; 2) minimally invasive esophagectomy (Ivor-Lewis or McKowen surgery); and 3) intraoperative indwelling of nasointestinal or jejunostomy tube. Exclusion criteria were: 1) incomplete clinical data; 2) conversion to open surgery intraoperatively; or 3) postoperative parenteral nutrition.

### Surgical methods

Patients underwent McKeown surgery to achieve esophagogastric anastomosis, as described previously [17]. During surgery, the surgeons placed the nasointestinal tube into the second lateral hole of the end of the gastric tube and introduced it from the nasal cavity, passing through the esophagus, residual stomach, and the pylorus, to reach the duodenum. Then, the surgeons grasped the bifurcation of the gastric tube and the duodenal nutrition tube in the abdominal cavity and removed the nutrition tube until the two were separated. Subsequently, the nutrition tube was further inserted by about 20 cm, while the gastric tube was withdrawn to enter the stomach. Routine indwelling of the nasointestinal tube was performed at an overall depth of about 75–80 cm away from incisors. After indwelling completion, the nasointestinal tube and the gastric tube were simultaneously fixed to the nose with adhesive plaster and a string.

The patients received Ivor-Lewi surgery [18, 19] to achieve intrathoracic esophagogastrostomy. After laparoscopic preparation of the tubular stomach and abdominal lymph node dissection, the jejunum was lifted 25 cm from the ligament of Treitz, and laparoscopic purse string suture was conducted with a 4–0 non-invasive suture; the fixation line was reserved at the area 0.5 cm away from the proximal end. Using the operating hole at the left lower abdomen as the ostomy hole, cauterization was performed with an electrotome, penetrating the intestinal wall. Then, a Flocare CH10–130 nutrition tube was placed into the distal end of the jejunum, and water was supplied while the nutrition tube was being placed into the intestinal tube, at a depth of 40 cm. After water injection was smooth, the purse string was tightened. The end of the string was inserted subcutaneously with a needle, and the jejunostomy tube was fixed to the abdominal wall with a No. 4 suture.

All patients in both groups discontinued enteral nutrition 3 weeks after surgery.

### Data collection

General clinical parameters (age, sex, tumor location, pTMN stage, pathological G stage, and previous history of abdominal surgery), perioperative data (surgery time, intraoperative indwelling time, anal exhaust time, postoperative hospital stay, hospitalization expenses, perioperative complications, and tubule-related complications), and postoperative long-term complications were assessed.

The primary outcome was perioperative complications. Postoperative long-term complications, tubule-related complications, postoperative indwelling time of nutrition tube, anal exhaust time, hospitalization expense, and postoperative hospital stay were secondary outcomes.

### Follow up

Outpatient follow up was performed at 1, 3, 6, 9, and 12 months after surgery. Routine blood tests, blood biochemistry, tumor biomarkers, esophagography, and chest and upper abdominal CT examinations were performed. In addition, the patients were evaluated for digestive symptoms, including abdominal pain, diarrhea, vomiting, acid reflux, and/or eating/swallowing obstruction. Patients unable to return to the outpatient center were followed up by phone, recording symptoms and results of examinations performed at local hospitals. The follow up was completed in December 2018.

### Statistical analysis

Data analysis was performed with the SPSS 22.0 statistical software (SPSS, USA). Quantitative data with normal distribution were expressed as mean ± SD and compared by Student’s t test. Categorical data were expressed as frequency and percentage and assessed by the χ^2^ and Wilcoxon rank sum tests for nominal and ordinal variables, respectively. *P* < 0.05 was considered statistically significant.

## Results

### Patient characteristics

A total of 1400 patients were enrolled in the present study. Of these, 1044 underwent minimally invasive Ivor-Lewis surgery, with routine jejunostomy; meanwhile, 356 underwent minimally invasive McKeown surgery, with routine indwelling nasal nutrition tubes. The median follow-up time was 8 months (1-12 months). 384 patients were lost to follow-up.

There were no significant differences in sex, age, preoperative ASA grade, postoperative TNM stage, pathological G stage, and previous history of abdominal surgery between the two groups (all *P* > 0.05). Regarding tumor location, the difference between the two groups was statistically significant (*P* < 0.001). All patients in the Jejunostomy group had middle or lower thoracic esophageal carcinoma; in the Nasojejunal group, 201, 147 and 8 patients were upper, middle and lower thoracic esophageal carcinoma cases, respectively. Among them, there were 8 cases of lower thoracic esophageal carcinoma with T1N0M0 complicated with preoperative severe lung function damage, including 5 and 3 administered esophagectomy and inflatable mediastinoscopy plus laparoscopic esophagectomy, respectively (Table 1).

**Table 1:** Patient baseline data

	Before match		
	Jejunostomy group (*n* = 1044)	Nasojejunal group (*n* = 356)	*P* value
Sex (Male)	817 (78.3%)	272 (76.4%)	0.514
Age (y)	61.8 ± 16.5	63.5 ± 14.6	0.084
Tumor location			< 0.001
Superior segment	0 (0.0%)	201 (56.5%)	
Middle segment	621 (59.5%)	147 (41.3%)	
Inferior segment	423 (40.5%)	8 (2.2%)	
Preoperative ASA staging			0.085
I	176 (16.9%)	53 (14.9%)	
II	725 (69.4%)	237 (66.6%)	
III	143 (13.7%)	66 (18.5%)	
Postoperative TNM staging			
Postoperative T staging			0.172
T1	103 (9.8%)	35 (9.8%)	
T2	216 (20.7%)	64 (18.0%)	
T3	624 (59.8%)	208 (58.4%)	
T4	101 (9.7%)	49 (13.8%)	
Postoperative N staging			0.127
N0	208 (19.9%)	54 (15.2%)	
N1	626 (60.0%)	228 (64.0%)	
N2	210 (20.1%)	74 (20.8%)	
Pathological G staging			0.649
G1	208 (19.9%)	71 (19.9%)	
G2	731 (70.0%)	243 (68.3%)	
G3	105 (10.1%)	42 (11.8%)	
Previous history of abdominal surgery	98 (9.4%)	31 (8.7%)	0.782

### Perioperative data and postoperative long-term outcomes

Perioperative and postoperative findings are summarized in Table 2. Operation time (208.8 ± 53.5 min vs. 218.1 ± 43.2 min) was shorter in the Jejunostomy group compared with the Nasojejunal group, while intraoperative (26.6 ± 10.4 min vs 18.4 ± 9.1 min) and postoperative (38.6 ± 6.9 min vs 18.5 ± 7.6 min) indwelling times of nutrition tubes were prolonged (all *P* < 0.05). There were no significant differences in hospitalization expenses, postoperative anal exhaust time, and postoperative hospital stay between the two groups (all *P* > 0.05). There were also no significant differences between the two groups regarding gastrointestinal/nasal hemorrhage, abdominal infection, intestinal fistula, anastomotic fistula, and perioperative mortality rates (all P > 0.05). Postoperative pulmonary infection (17.0% vs 22.2%), incision infection (0.2% vs 1.1%), nutrient tube slippage (0.2% vs 5.1%) and nutrient reflux 1 (0.1% vs 5.6%) rates were reduced in the Jejunostomy group compared with the Nasojejunal group (*P* < 0.05). In all, there were 18 cases with nutrient tube slippage after surgery in the Nasojejunal group, and all reported extubation without autonomous consciousness at night; tube indwelling was performed again under fluoroscopy after extubation. There was 1 patient with sudden acute cardiopulmonary arrest in the Jejunostomy group, who was transferred to the ICU for further treatment after successful rescue; the patient was discharged upon request from family members.

**Table 2:** Perioperative clinical data and postoperative long-term conditions

	Before match		
	Jejunostomy group (n = 1044)	Nasojejunal group (n = 356)	*P* value
Surgery time (min)	208.8 ± 53.5	218.1 ± 43.2	0.003
Intraoperative indwelling time (min)	26.6 ± 10.4	18.4 ± 9.1	< 0.001
Postoperative indwelling time of nutrition tube (d)	38.6 ± 6.9	18.5 ± 7.6	< 0.001
Anal exhaust time (d)	2.6 ± 2.3	3.0 ± 1.3	0.066
Hospitalization expense (ten thousand yuan)	5.3 ± 2.4	5.6 ± 4.0	0.103
Postoperative hospital stay (d)	14.8 ± 5.8	15.5 ± 6.1	0.088
Perioperative complications (n)			
Pulmonary infection	178 (17.0%)	79 (22.2%)	0.037
Ileus	18 (1.7%)	1 (0.3%)	0.042
Abdominal infection	2 (0.2%)	2 (0.6%)	0.258
Anastomotic fistula	36 (3.5%)	15 (4.2%)	0.616
Perioperative death	1 (0.1%)	0 (0.0%)	0.559
Incision infection	2 (0.2%)	4 (1.1%)	0.020
Tubule-related complications (n)			
Gastrointestinal/nasal hemorrhage	11 (1.1%)	4 (1.1%)	0.912
Nutrient tube slippage	2 (0.2%)	18 (5.1%)	< 0.001
Intestinal fistula	8 (0.8%)	0 (0.0%)	0.098
Nutrient reflux	1 (0.1%)	20 (5.6%)	< 0.001
Postoperative long-term complications (> 3 m, n)			
Ileus	18 (1.7%)	1 (0.3%)	0.042

Meanwhile, ileus rates perioperatively (1.7% vs 0.3%) and at 3 postoperative months (1.7% vs 0.3%) were both higher in the Jejunostomy group compared with the Nasojejunal group. Precisely, there were 18 patients with postoperative ileus in the Jejunostomy group; all those with incomplete ileus who had a previous history of abdominal surgery were improved after parenteral nutrition support and discontinuation of nasal feeding. There were 18 cases with ileus 3 months after surgery, including 17 who had incomplete ileus and were improved after conservative treatment; 1 case was improved after separation of intestinal adhesion.

## Discussion

In this study, operation time was shorter in the Jejunostomy group compared with the Nasojejunal group, while intraoperative and postoperative indwelling times of nutrition tubes were prolonged, demonstrating superior treatment effects for jejunostomy; meanwhile, adverse effects were generally less frequent after jejunostomy.

Further development of surgical treatment in esophageal carcinoma aims to reduce surgical trauma, and improve postoperative quality of life, as well as short- and long-term efficacies. Postoperative EN in patients with esophageal carcinoma plays an important role in maintaining normal body metabolism, functional recovery, reducing complications, and hospitalization expenses [8, 20]. With continuous improvement of laparoscopic equipment and techniques, laparoscopic jejunostomy has also been gradually introduced in minimally invasive esophagectomy in recent years. Matching the apparatus and nutrition tubes in jejunostomy would certainly increase the economic burden to some extent. In response, our center has performed laparoscopic jejunostomy since 2015, and routine laparoscopic equipment and common nutrition tubes have been used, which has reduced hospitalization expenses while avoiding excessive dependence on special apparatus in further popularization. In the present study, 1400 patients undergoing minimally invasive esophagectomy were assessed, and jejunostomy had the advantages of reducing postoperative pulmonary infection, nutrient tube slippage, nutrient reflux, and incision infection. Although the incidence of perioperative ileus and postoperative long-term ileus were slightly higher than those of the Nasojejunal group, they were relatively low, which was acceptable.

The above data showed that the Jejunostomy group was superior to the Nasojejunal group in terms of surgery time, postoperative indwelling time of the nutrition tube, postoperative pulmonary infection, and postoperative incision infection, although ileus rates were relatively higher. Zhao Song et al. [21] conducted a retrospective comparative analysis of different EN methods in 128 patients with esophageal carcinoma, and reported prolonged surgery time and postoperative indwelling time of the nutrition tube in the jejunostomy group compared with the nasojejunal group, indicating that jejunostomy improves the nutritional status of patients in a better way. The incidence of postoperative complications (7.69%) in the jejunostomy group was significantly higher than that of the nasojejunal group (26.31%), and it was considered that the nasointestinal tube stimulated the nasopharynx, causing difficulty in coughing, expectoration and intestinal nutrient reflux, which are main factors exacerbating pulmonary infections. Wang et al. [22] analyzed the clinical data of 28 patients with esophageal carcinoma administered laparoscopic jejunostomy, showing that the nasointestinal tube easily results in nutrient reflux, causing vomiting and aspiration pneumonia, with poor patient tolerance. Meanwhile, the advantages of a jejunostomy tube are as follows: it can be carried out a long time after surgery; when complications, such as anastomotic fistula, occur, the patients can receive long-term EN to improve prognosis; patients have high tolerance with no obvious foreign body sensation, resulting in limited effects on daily life activities. Rong Baolin et al. [23] analyzed the clinical data of 279 patients with esophageal carcinoma administered laparoscopic jejunostomy at an earlier period in our hospital, and suggested a total incidence of postoperative complications of 20.1%, including 4 patients with incomplete ileus, 1 with complete ileus 3 months after surgery, and 1 with angulated torsion of the intestinal tube in the jejunostomy region shown in secondary surgical exploration, who recovered after releasing the intestinal adhesion. It is believed that the Nasojejunal group achieves cervical anastomosis, and the incidence of damage to the recurrent laryngeal nerve is higher compared with that of intrathoracic anastomosis; in addition, the nasointestinal tube is indwelt through the nose, causing cough, expectoration, nausea, and reflux, thus increasing the incidence of pulmonary infection to some extent [13]. Meanwhile, all incisions in the Jejunostomy group are of laparoscopic nature, and small auxiliary incisions are present at the upper abdomen and neck in the Nasojejunal group, so the incidence of incision infection is relatively high. In addition, a jejunostomy tube looks better and is more convenient than a nasointestinal tube, avoiding negative effects of a nasointestinal tube on social function after discharge and reducing feelings of inferiority caused by the disease.

Although the incidence of postoperative ileus was higher after jejunostomy than in the Nasojejunal group, the overall rate was not very high, and the involved patients had a history of abdominal surgery. It is recommended that patients with a previous history of abdominal surgery should be treated with tube feeding only after confirming no ileus by abdominal imaging and patients with postoperative abdominal distension and pain should be further examined. Nasal feeding should be discontinued for those with postoperative ileus, avoiding further aggravation of their condition.

Tubule-related complications are among the main evaluation factors for postoperative EN, which mainly include intestinal fistula, nutrient tube slippage, tube blocking, and ileus [13, 15, 24]. The jejunostomy tube reported in most studies has a relatively large diameter. Although this decreases the rate of tube blockage, other tubule-related complications can increase. Zhou et al. [24] analyzed the clinical data of 42 patients with esophageal carcinoma between January 2014 and July 2015, and the main tubule-related complication after jejunostomy was intestinal fistula, which may be due to loose knotting of the purse string. Postoperative care after jejunostomy was very important in preventing tube slippage and blocking. Sun Ming et al. [13] retrospectively analyzed EN in 90 patients undergoing esophagectomy through two incisions at the right chest and abdomen, showing the nasojejunal group had different degrees of nasopharyngeal discomfort; in addition, incidence of tube slippage and blocking were significantly lower in the jejunostomy group compared with the nasojejunal group. This demonstrates that although nasal feeding via a nasointestinal tube is simple, easy, and suitable for short-term feeding, it has certain shortcomings: it may damage the anastomotic stoma or stump during tube indwelling, which may easily cause intraoperative contamination; some patients have nutrient reflux during nasal feeding, easily resulting in lung infection and anastomotic contamination; the nasointestinal tube is generally fixed with tape and rope, and some patients may remove it due to nasopharyngeal discomfort, leading to nutrient tube slippage. Gong et al. [15] analyzed tubule-related complications in 379 patients with esophageal carcinoma administered jejunostomy between 2005 and 2013, showing that approximately 13–40% of individuals had tubule-related complications, mainly including swelling and infection around the jejunostomy area, intestinal fistula, and tube blocking; therefore, they recommended properly reducing the proportion of jejunostomy for patients with early stage of tumor and no need for further treatment after surgery, and postoperative EN treatment via a nasointestinal tube instead. While jejunostomy can be performed in patients with advanced-stage tumors, and long-term indwelling would improve the nutritional status. However, it is important to note that the diameters of the jejunostomy and nasointestinal tubes reported in the above studies were different, with certain effects on the results of postoperative tubule-related complications. In the present study, patients in the two groups were treated with Flocare CH 10–130 nutrition tube (Nutricia pharmaceutical co. LTD, Wuxi, China). There was no significant difference in the rate of tube blocking between the two groups, which may be predominantly related to product information, education, and nursing regarding the nutrition tube. The incidence of intestinal fistula and ileus in the Jejunostomy group were higher than those of the Nasojejunal group. Meanwhile, the Jejunostomy group showed lower nutrient reflux and nutrient tube slippage rates than the Nasojejunal group. This indicates that although the nasointestinal tube was used for a long time in esophagectomy with high safety, tubule-related complications were relatively high, and patient comfort was poor; therefore, it was mainly applied for EN after McKeown surgery. With the widespread application of minimally invasive esophagectomy in recent years, especially the popularity of minimally invasive Ivor-Lewis surgery, a traditional nasointestinal tube is no longer suitable for laparoscopic surgery and laparoscopic jejunostomy is more suitable for minimally invasive Ivor-Lewis surgery. Although certain surgical trauma and postoperative complications will occur, most complications could be further prevented and avoided with the accumulation of related experience.

This study had several limitations. It was a single-center retrospective trial with a certain bias in case selection. For example, we could not perform a propensity-score matched analysis to compare the outcomes between the two matched groups because too many patients in the Nasojejunal group would have to be removed from the analysis because of the differences in cancer location between the groups. Although clinical effectiveness was significant, multi-center prospective studies are still needed for further investigation. The patient’s subjective tolerance conditions were not very detailed in the medical records, including nasopharyngeal discomfort, abdominal discomfort, nausea, vomiting, and number of extubations due to intolerance. Therefore, this study could not assess differences between the two enteral nutrition methods in that respect. In addition, patients used several types of enteral nutrients, including enteral nutrition suspensions and our in-house configured nutrients. Furthermore, this study failed to analyze nutrient differences between the two groups, because data of albumin and hemoglobin levels before surgery and at discharge were incomplete. Most importantly, patients in the two groups had different surgical methods. However, for the benefit of patients, the choice of the EN method would be affected by the surgical technique, so the influence of the surgical method on the above results could not be completely eliminated.

## Conclusion

In conclusion, jejunostomy is safe and reliable in the treatment of thoracic segment esophageal carcinoma during complete thoracoscopic and laparoscopic Ivor-Lewis esophagectomy. Compared with a nasointestinal tube in minimally invasive McKeown surgery, it has the advantages of reducing postoperative pulmonary infection, nutrient tube slippage, nutrient reflux, and incision infection. Although the incidence of perioperative ileus and postoperative long-term ileus were slightly higher than those of the Nasojejunal group, the overall rates were relatively low and within an acceptable range. Further accumulation of related experience and techniques would help prevent and treat these postoperative complications in the future.

## Data Availability

The datasets used and/or analyzed during the current study are available from the corresponding author on reasonable request.

## Abbreviations

NCCN: National Comprehensive Cancer Network
EN: enteral nutrition
